# Diaqua­bis­(*N*,*N*-diethyl­nicotinamide-κ*N* ^1^)bis­(4-ethyl­benzoato-κ*O*)cobalt(II)

**DOI:** 10.1107/S1600536811014188

**Published:** 2011-04-22

**Authors:** Hacali Necefoğlu, Ali Maracı, Füreya Elif Özbek, Barış Tercan, Tuncer Hökelek

**Affiliations:** a Department of Chemistry, Kafkas University, 36100 Kars, Turkey; b Department of Physics, Karabük University, 78050, Karabük, Turkey; c Department of Physics, Hacettepe University, 06800 Beytepe, Ankara, Turkey

## Abstract

The title Co^II^ complex, [Co(C_9_H_9_O_2_)_2_(C_10_H_14_N_2_O)_2_(H_2_O)_2_], contains two 4-ethyl­benzoate (PEB), two monodentate diethyl­nicotinamide (DENA) ligands and two water mol­ecules. The four O atoms in the equatorial plane around the Co^II^ ion form a slightly distorted square-planar arrangement, while the slightly distorted octa­hedral coordination is completed by the two N atoms of the DENA ligands in the axial positions. Intra­molecular O—H⋯O hydrogen bonds link the water mol­ecules to the carboxyl­ate groups. The dihedral angles between the carboxyl­ate groups and the adjacent benzene rings are 4.52 (18) and 4.56 (18)°, while the pyridine rings and the benzene rings are oriented at dihedral angles of 7.76 (10) and 5.67 (13)°. In the crystal, inter­molecular O—H⋯O hydrogen bonds link the mol­ecules into chains propagating along [010]. C—H⋯O inter­actions and a π–π contact between the pyridine rings [centroid–centroid distance = 3.476 (2) Å] are also observed.

## Related literature

For background to niacin, see: Krishnamachari (1974 ▶) and to the nicotinic acid derivative *N*,*N*-diethyl­nicotinamide, see: Bigoli *et al.* (1972 ▶). For related structures, see: Hökelek *et al.* (1996 ▶); Hökelek & Necefoğlu (1998 ▶, 2007 ▶); Hökelek *et al.* (2009*a* ▶,*b* ▶). For bond-length data, see: Allen *et al.* (1987 ▶).
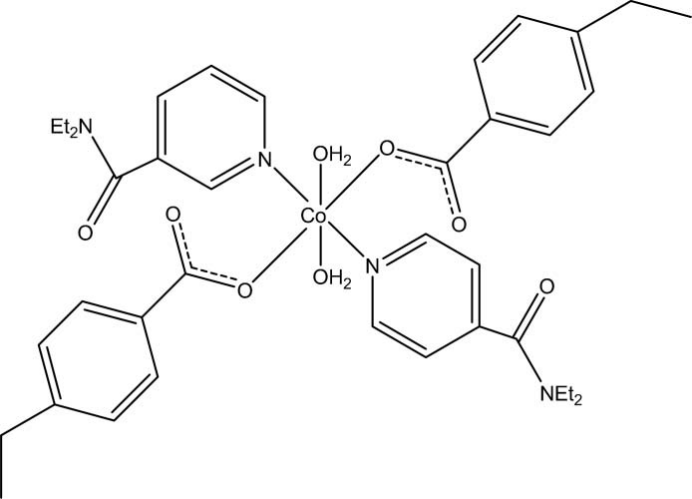


## Experimental

### 

#### Crystal data

[Co(C_9_H_9_O_2_)_2_(C_10_H_14_N_2_O)_2_(H_2_O)_2_]
*M* *_r_* = 749.75
Monoclinic, 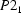

*a* = 8.4292 (2) Å
*b* = 11.9399 (3) Å
*c* = 18.1716 (4) Å
β = 98.685 (3)°
*V* = 1807.89 (8) Å^3^
*Z* = 2
Mo *K*α radiation
μ = 0.53 mm^−1^
*T* = 100 K
0.35 × 0.23 × 0.19 mm

#### Data collection

Bruker Kappa APEXII CCD area-detector diffractometer
Absorption correction: multi-scan (*SADABS*; Bruker, 2005) ▶ *T*_min_ = 0.862, *T*_max_ = 0.902
27964 measured reflections
6307 independent reflections
5116 reflections with *I* > 2σ(*I*)
*R*_int_ = 0.056

#### Refinement

*R*[*F* ^2^ > 2σ(*F* ^2^)] = 0.044
*wR*(*F* ^2^) = 0.095
*S* = 1.02
6307 reflections
479 parameters
5 restraints
H atoms treated by a mixture of independent and constrained refinement
Δρ_max_ = 0.97 e Å^−3^
Δρ_min_ = −0.28 e Å^−3^
Absolute structure: Flack (1983 ▶), 2955 Friedel pairs
Flack parameter: 0.371 (13)

### 

Data collection: *APEX2* (Bruker, 2007 ▶); cell refinement: *SAINT* (Bruker, 2007 ▶); data reduction: *SAINT*; program(s) used to solve structure: *SHELXS97* (Sheldrick, 2008 ▶); program(s) used to refine structure: *SHELXL97* (Sheldrick, 2008 ▶); molecular graphics: *ORTEP-3 for Windows* (Farrugia, 1997 ▶); software used to prepare material for publication: *WinGX* (Farrugia, 1999 ▶) and *PLATON* (Spek, 2009 ▶).

## Supplementary Material

Crystal structure: contains datablocks I, global. DOI: 10.1107/S1600536811014188/su2268sup1.cif

Structure factors: contains datablocks I. DOI: 10.1107/S1600536811014188/su2268Isup2.hkl

Additional supplementary materials:  crystallographic information; 3D view; checkCIF report

## Figures and Tables

**Table 1:** Hydrogen-bond geometry (Å, °)

*D*—H⋯*A*	*D*—H	H⋯*A*	*D*⋯*A*	*D*—H⋯*A*
O7—H71⋯O6^i^	0.84 (3)	2.02 (4)	2.767 (4)	148 (3)
O7—H72⋯O4	0.85 (2)	1.79 (2)	2.622 (3)	167 (4)
O8—H81⋯O4^ii^	0.83 (4)	1.98 (3)	2.811 (3)	176 (4)
O8—H82⋯O2	0.86 (2)	1.77 (2)	2.614 (3)	167 (5)
C15—H15⋯O5^iii^	0.95	2.40	3.170 (5)	138
C20—H20⋯O4^iii^	0.95	2.49	3.407 (4)	163
C30—H30⋯O6^iv^	0.95	2.34	3.262 (4)	163

Symmetry codes: (i) 
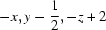
; (ii) 
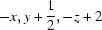
; (iii) 
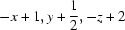
; (iv) 
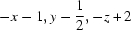
.

**Fig. 1.**
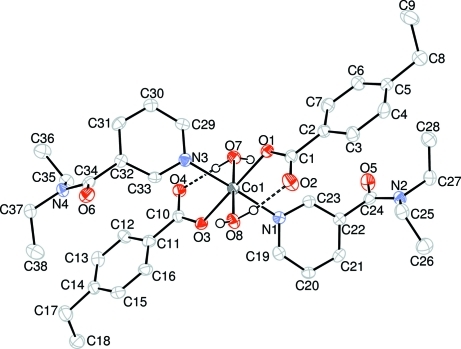
The molecular structure of the title molecule with the atom-numbering scheme. Displacement ellipsoids are drawn at the 50% probability level. The intramolecular O-H···O hydrogen bonds are shown as dashed lines.

**Fig. 2.**
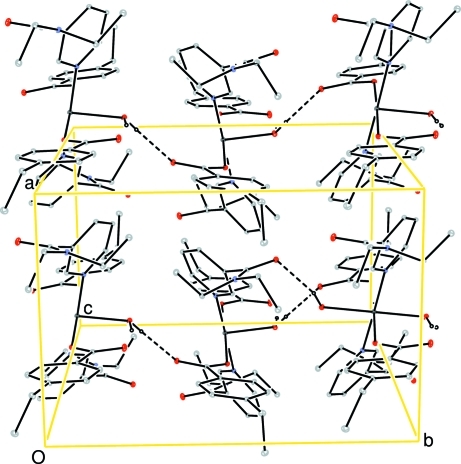
A view of the crystal packing of the title compound. The O-H···O hydrogen bonds are shown as dashed lines [see Table 1 for details; H-atoms not involved in hydrogen bonding have been omitted for clarity].

## supplementary crystallographic information

### Comment

As a part of our ongoing investigations of transition metal complexes of nicotinamide (NA), one form of niacin (Krishnamachari, 1974), and/or the nicotinic acid derivative *N*,*N*-diethylnicotinamide (DENA), an important respiratory stimulant (Bigoli *et al.*, 1972), the title compound was synthesized and its crystal structure is reported on herein.

The title mononuclear Co^II^complex, (Fig. 1), consisting of two *N*,*N*-diethylnicotinamide (DENA), two 4-ethylbenzoate (PEB) ligands and two coordinated water molecules, all ligands coordinating in a monodentate manner. The crystal structures of similar complexes of Cu^II^, Co^II^, Ni^II^, Mn^II^ and Zn^II^ ions, [Cu(C_7_H_5_O_2_)_2_(C_10_H_14_N_2_O)_2_] (Hökelek *et al.*, 1996), [Co(C_6_H_6_N_2_O)_2_(C_7_H_4_NO_4_)_2_(H_2_O)_2_] (Hökelek & Necefoğlu, 1998), [Ni(C_7_H_4_ClO_2_)_2_(C_6_H_6_N_2_O)_2_(H_2_O)_2_] (Hökelek *et al.*, 2009*a*), [Mn(C_9_H_10_NO_2_)_2_(H_2_O)_4_].2H_2_O (Hökelek & Necefoğlu, 2007) and [Zn(C_7_H_4_BrO_2_)_2_(C_6_H_6_N_2_O)_2_(H_2_O)_2_] (Hökelek *et al.*, 2009*b*), have also been reported. In the copper(II) complex mentioned above the two benzoate ions coordinate to the Cu^II^ atom as bidentate ligands, while in the other structures all the ligands coordinate in a monodentate manner.

In the title complex, the four O atoms (O1, O3, O7 and O8) in the equatorial plane around the Co^II^ ion form a slightly distorted square-planar arrangement, while the slightly distorted octahedral coordination is completed by the two N atoms of the DENA ligands (N1 and N3) in the axial positions. Intramolecular O-H···O hydrogen bonds link the water molecules to the carboxylate groups (Table 1 and Fig. 1). The near equalities of the C1—O1 [1.252 (4) Å], C1—O2 [1.245 (4) Å] and C10—O3 [1.262 (4) Å], C10—O4 [1.243 (4) Å] bonds in the carboxylate groups indicate delocalized bonding arrangements, rather than localized single and double bonds. The Co—O bond lengths are 2.057 (2) and 2.055 (2) Å (for benzoate oxygens) and 2.117 (3) and 2.114 (3) Å (for water oxygens), and the Co—N bond lengths are 2.118 (3) and 2.120 (3) Å, close to standard values (Allen *et al.*, 1987). The Co atom is displaced out of the mean-planes of the carboxylate groups (O1/C1/O2) and (O3/C10/O4) by -0.7356 (4) and 0.8040 (4) Å, respectively. The dihedral angles between the planar carboxylate groups and the adjacent benzene rings A (C2—C7) and B (C11—C16) are 4.52 (18) and 4.62 (18) °, respectively. The benzene A (C2—C7) and B (C11—C16) rings and the pyridine C (N1/C19—C23) and D (N3/C29—C33) rings are oriented at dihedral angles of A/B = 5.67 (13), A/C = 63.76 (13), A/D = 58.10 (13), B/C = 59.21 (12), B/D = 53.96 (12) and C/D = 7.76 (10) °.

In the crystal, intermolecular O—H···O hydrogen bonds link the molecules into chains propagating along [010] (Table 1 and Fig. 2). There also exist C-H···O interactions leading to the formation of two-dimensional networks lying parallel to (110). The π–π contact between the pyridine rings, Cg3—Cg4^i^, may further stabilize the crystal structure [centroid-to-centroid distance = 3.476 (2) Å; symmetry code: (i) x - 1, y, z; Cg3 and Cg4 are the centroids of the rings C (N1/C19—C23) and D (N3/C29—C33), respectively].

### Experimental

The title compound was prepared by the reaction of CoSO_4_.7H_2_O (1.40 g, 5 mmol) in H_2_O (100 ml) and *N*,*N*-diethylnicotinamide (1.78 g, 10 mmol) in H_2_O (50 ml) with sodium 4-ethylbenzoate (1.72 g, 10 mmol) in H_2_O (100 ml) at room temperature. The mixture was filtered and set aside to crystallize at ambient temperature for four days, giving pink single crystals.

### Refinement

The compound crystallized as an inversion twin: refined BASF parameter = 0.371 (13), for 2995 Friedel pairs (88.2% coverage). The H-atoms of the water molecules (H71, H72, H81 and H82) were located in a difference Fourier map and were freely refined. The C-bound H-atoms were positioned geometrically with C—H = 0.93, 0.97 and 0.96 Å, for aromatic, methylene and methyl H-atoms, respectively, and constrained to ride on their parent atoms, with *U*_iso_(H) = k × *U*_eq_(C), where k = 1.5 for methyl H-atoms and k = 1.2 for all other H-atoms.

### Crystal data

**Table:**

[Co(C_9_H_9_O_2_)_2_(C_10_H_14_N_2_O)_2_(H_2_O)_2_]	*F*(000) = 794
*M**_r_* = 749.75	*D*_x_ = 1.377 Mg m^−^^3^
Monoclinic, *P*2_1_	Mo *K*α radiation, λ = 0.71073 Å
Hall symbol: P 2yb	Cell parameters from 6030 reflections
*a* = 8.4292 (2) Å	θ = 2.4–23.6°
*b* = 11.9399 (3) Å	µ = 0.53 mm^−^^1^
*c* = 18.1716 (4) Å	*T* = 100 K
β = 98.685 (3)°	Block, pink
*V* = 1807.89 (8) Å^3^	0.35 × 0.23 × 0.19 mm
*Z* = 2	

### Data collection

**Table:**

Bruker Kappa APEXII CCD area-detector diffractometer	6307 independent reflections
Radiation source: fine-focus sealed tube	5116 reflections with *I* > 2σ(*I*)
graphite	*R*_int_ = 0.056
φ and ω scans	θ_max_ = 25.0°, θ_min_ = 2.1°
Absorption correction: multi-scan (*SADABS*; Bruker, 2005)	*h* = −10→9
*T*_min_ = 0.862, *T*_max_ = 0.902	*k* = −14→13
27964 measured reflections	*l* = −21→21

### Refinement

**Table:**

Refinement on *F*^2^	Secondary atom site location: difference Fourier map
Least-squares matrix: full	Hydrogen site location: inferred from neighbouring sites
*R*[*F*^2^ > 2σ(*F*^2^)] = 0.044	H atoms treated by a mixture of independent and constrained refinement
*wR*(*F*^2^) = 0.095	*w* = 1/[σ^2^(*F*_o_^2^) + (0.0486*P*)^2^] where *P* = (*F*_o_^2^ + 2*F*_c_^2^)/3
*S* = 1.02	(Δ/σ)_max_ < 0.001
6307 reflections	Δρ_max_ = 0.97 e Å^−^^3^
479 parameters	Δρ_min_ = −0.28 e Å^−^^3^
5 restraints	Absolute structure: Flack (1983), 2955 Friedel pairs
Primary atom site location: structure-invariant direct methods	Flack parameter: 0.371 (13)

### Special details

**Table:**

Geometry. All esds (except the esd in the dihedral angle between two l.s. planes) are estimated using the full covariance matrix. The cell esds are taken into account individually in the estimation of esds in distances, angles and torsion angles; correlations between esds in cell parameters are only used when they are defined by crystal symmetry. An approximate (isotropic) treatment of cell esds is used for estimating esds involving l.s. planes.
Refinement. Refinement of F^2^ against ALL reflections. The weighted R-factor wR and goodness of fit S are based on F^2^, conventional R-factors R are based on F, with F set to zero for negative F^2^. The threshold expression of F^2^ > 2sigma(F^2^) is used only for calculating R-factors(gt) etc. and is not relevant to the choice of reflections for refinement. R-factors based on F^2^ are statistically about twice as large as those based on F, and R- factors based on ALL data will be even larger.

### Fractional atomic coordinates and isotropic or equivalent isotropic displacement parameters (Å^2^)

**Table:**

	*x*	*y*	*z*	*U*_iso_*/*U*_eq_	
Co1	0.09357 (5)	0.99959 (4)	0.90987 (2)	0.02139 (13)	
O1	−0.0091 (2)	0.9900 (2)	0.79991 (11)	0.0222 (5)	
O2	0.0048 (3)	1.1657 (2)	0.76125 (12)	0.0340 (7)	
O3	0.2009 (2)	1.0035 (2)	1.01919 (10)	0.0230 (5)	
O4	0.1608 (3)	0.83109 (19)	1.05747 (13)	0.0259 (6)	
O5	0.5443 (3)	0.8411 (2)	0.71932 (13)	0.0347 (7)	
O6	−0.3650 (3)	1.1694 (2)	1.09285 (13)	0.0288 (6)	
O7	0.1212 (3)	0.8233 (2)	0.91162 (13)	0.0270 (7)	
H71	0.201 (4)	0.794 (3)	0.897 (2)	0.061*	
H72	0.141 (5)	0.816 (4)	0.9586 (11)	0.061*	
O8	0.0658 (3)	1.1756 (2)	0.90649 (13)	0.0270 (7)	
H81	−0.005 (4)	1.219 (3)	0.917 (2)	0.061*	
H82	0.047 (5)	1.183 (4)	0.8591 (11)	0.061*	
N1	0.3167 (3)	1.0292 (2)	0.87320 (14)	0.0213 (7)	
N2	0.5169 (3)	1.0013 (3)	0.65219 (13)	0.0246 (6)	
N3	−0.1239 (3)	0.9685 (2)	0.95161 (14)	0.0230 (7)	
N4	−0.3059 (3)	1.0308 (2)	1.17585 (15)	0.0234 (7)	
C1	−0.0075 (4)	1.0629 (3)	0.75050 (18)	0.0227 (9)	
C2	−0.0195 (4)	1.0222 (3)	0.67217 (18)	0.0223 (8)	
C3	−0.0240 (5)	1.0975 (3)	0.61439 (19)	0.0267 (9)	
H3	−0.0202	1.1756	0.6245	0.032*	
C4	−0.0338 (5)	1.0604 (3)	0.5420 (2)	0.0314 (10)	
H4	−0.0409	1.1133	0.5026	0.038*	
C5	−0.0335 (5)	0.9470 (3)	0.5261 (2)	0.0262 (9)	
C6	−0.0268 (5)	0.8739 (3)	0.5843 (2)	0.0284 (10)	
H6	−0.0267	0.7957	0.5745	0.034*	
C7	−0.0203 (4)	0.9098 (3)	0.65583 (19)	0.0269 (9)	
H7	−0.0163	0.8565	0.6949	0.032*	
C8	−0.0481 (5)	0.9055 (3)	0.44706 (19)	0.0348 (9)	
H8A	−0.0338	0.9693	0.4139	0.042*	
H8B	0.0386	0.8508	0.4433	0.042*	
C9	−0.2068 (5)	0.8513 (4)	0.4212 (2)	0.0416 (11)	
H9A	−0.2087	0.8226	0.3705	0.062*	
H9B	−0.2927	0.9065	0.4216	0.062*	
H9C	−0.2228	0.7892	0.4546	0.062*	
C10	0.1872 (4)	0.9325 (3)	1.06922 (18)	0.0220 (9)	
C11	0.2042 (4)	0.9771 (3)	1.14683 (18)	0.0256 (9)	
C12	0.1993 (4)	0.9059 (3)	1.20641 (19)	0.0275 (9)	
H12	0.1864	0.8275	1.1985	0.033*	
C13	0.2134 (5)	0.9491 (3)	1.2770 (2)	0.0294 (10)	
H13	0.2105	0.8998	1.3178	0.035*	
C14	0.2316 (5)	1.0629 (3)	1.2902 (2)	0.0267 (10)	
C15	0.2334 (5)	1.1324 (3)	1.2299 (2)	0.0303 (10)	
H15	0.2447	1.2109	1.2376	0.036*	
C16	0.2192 (4)	1.0910 (3)	1.15974 (19)	0.0276 (9)	
H16	0.2197	1.1408	1.1190	0.033*	
C17	0.2558 (5)	1.1087 (4)	1.36762 (19)	0.0382 (10)	
H17A	0.1985	1.0606	1.3993	0.046*	
H17B	0.2082	1.1846	1.3669	0.046*	
C18	0.4295 (5)	1.1154 (3)	1.4012 (2)	0.0385 (10)	
H18A	0.4382	1.1411	1.4529	0.058*	
H18B	0.4850	1.1682	1.3726	0.058*	
H18C	0.4787	1.0411	1.4000	0.058*	
C19	0.4216 (4)	1.1017 (3)	0.90860 (18)	0.0244 (8)	
H19	0.3978	1.1358	0.9529	0.029*	
C20	0.5630 (4)	1.1294 (3)	0.88390 (18)	0.0214 (8)	
H20	0.6356	1.1811	0.9107	0.026*	
C21	0.5967 (4)	1.0806 (3)	0.81971 (18)	0.0238 (8)	
H21	0.6929	1.0986	0.8010	0.029*	
C22	0.4896 (4)	1.0052 (3)	0.78262 (15)	0.0200 (7)	
C23	0.3534 (4)	0.9813 (3)	0.81233 (17)	0.0226 (8)	
H23	0.2813	0.9273	0.7878	0.027*	
C24	0.5188 (4)	0.9427 (3)	0.7153 (2)	0.0255 (9)	
C25	0.4561 (4)	1.1144 (3)	0.64169 (19)	0.0307 (10)	
H25A	0.4013	1.1352	0.6842	0.037*	
H25B	0.3759	1.1175	0.5959	0.037*	
C26	0.5879 (5)	1.1981 (3)	0.6354 (2)	0.0369 (10)	
H26A	0.5420	1.2735	0.6294	0.055*	
H26B	0.6397	1.1796	0.5921	0.055*	
H26C	0.6676	1.1953	0.6806	0.055*	
C27	0.5462 (4)	0.9410 (3)	0.58603 (19)	0.0296 (9)	
H27A	0.6227	0.8793	0.6009	0.036*	
H27B	0.5961	0.9925	0.5534	0.036*	
C28	0.3942 (5)	0.8935 (3)	0.54310 (19)	0.0365 (10)	
H28A	0.4188	0.8555	0.4984	0.055*	
H28B	0.3178	0.9543	0.5286	0.055*	
H28C	0.3471	0.8398	0.5744	0.055*	
C29	−0.2234 (4)	0.8863 (3)	0.92454 (18)	0.0233 (8)	
H29	−0.2010	0.8470	0.8818	0.028*	
C30	−0.3550 (4)	0.8555 (3)	0.95485 (19)	0.0255 (9)	
H30	−0.4221	0.7962	0.9337	0.031*	
C31	−0.3892 (4)	0.9120 (3)	1.01682 (19)	0.0250 (9)	
H31	−0.4799	0.8921	1.0394	0.030*	
C32	−0.2888 (4)	0.9981 (4)	1.04537 (15)	0.0202 (7)	
C33	−0.1595 (4)	1.0234 (3)	1.01041 (16)	0.0199 (8)	
H33	−0.0919	1.0836	1.0295	0.024*	
C34	−0.3228 (4)	1.0717 (3)	1.1070 (2)	0.0222 (8)	
C35	−0.2348 (4)	0.9213 (3)	1.19636 (19)	0.0282 (9)	
H35A	−0.1505	0.9298	1.2402	0.034*	
H35B	−0.1836	0.8920	1.1547	0.034*	
C36	−0.3591 (5)	0.8388 (3)	1.2142 (2)	0.0359 (10)	
H36A	−0.3072	0.7670	1.2289	0.054*	
H36B	−0.4403	0.8279	1.1702	0.054*	
H36C	−0.4104	0.8678	1.2553	0.054*	
C37	−0.3434 (4)	1.1026 (3)	1.23534 (18)	0.0271 (9)	
H37A	−0.3876	1.0562	1.2726	0.033*	
H37B	−0.4272	1.1569	1.2146	0.033*	
C38	−0.1988 (5)	1.1659 (3)	1.2735 (2)	0.0410 (10)	
H38A	−0.2314	1.2158	1.3113	0.061*	
H38B	−0.1525	1.2102	1.2366	0.061*	
H38C	−0.1186	1.1126	1.2973	0.061*	

### Atomic displacement parameters (Å^2^)

**Table:**

	*U*^11^	*U*^22^	*U*^33^	*U*^12^	*U*^13^	*U*^23^
Co1	0.0219 (2)	0.0213 (2)	0.0211 (2)	−0.0001 (2)	0.00336 (17)	−0.0003 (2)
O1	0.0223 (13)	0.0214 (13)	0.0232 (11)	−0.0008 (13)	0.0038 (10)	0.0008 (12)
O2	0.0556 (19)	0.0192 (15)	0.0271 (14)	0.0037 (13)	0.0056 (13)	0.0014 (12)
O3	0.0207 (12)	0.0257 (12)	0.0221 (11)	−0.0009 (14)	0.0011 (9)	0.0013 (13)
O4	0.0310 (16)	0.0179 (14)	0.0292 (14)	−0.0011 (11)	0.0055 (11)	−0.0008 (11)
O5	0.0480 (18)	0.0236 (15)	0.0349 (15)	0.0085 (13)	0.0143 (13)	0.0030 (11)
O6	0.0269 (15)	0.0217 (15)	0.0390 (15)	0.0002 (12)	0.0088 (12)	−0.0009 (12)
O7	0.0288 (17)	0.0248 (16)	0.0272 (16)	0.0014 (12)	0.0040 (14)	−0.0023 (11)
O8	0.0336 (17)	0.0260 (16)	0.0216 (15)	0.0020 (12)	0.0047 (14)	−0.0011 (11)
N1	0.0216 (17)	0.0204 (18)	0.0214 (15)	0.0021 (12)	0.0019 (13)	0.0009 (12)
N2	0.0235 (15)	0.0274 (15)	0.0236 (14)	0.0040 (17)	0.0058 (12)	−0.0044 (17)
N3	0.0224 (17)	0.026 (2)	0.0201 (15)	0.0010 (13)	0.0020 (13)	0.0009 (12)
N4	0.0277 (18)	0.0232 (19)	0.0199 (15)	0.0022 (13)	0.0055 (13)	0.0005 (12)
C1	0.019 (2)	0.027 (2)	0.023 (2)	0.0006 (16)	0.0042 (16)	−0.0001 (16)
C2	0.0188 (19)	0.026 (2)	0.0222 (18)	−0.0005 (16)	0.0022 (14)	0.0001 (15)
C3	0.032 (2)	0.024 (2)	0.025 (2)	0.0002 (18)	0.0063 (19)	0.0013 (18)
C4	0.034 (3)	0.036 (3)	0.026 (2)	−0.0054 (19)	0.0094 (19)	0.0072 (18)
C5	0.027 (2)	0.029 (2)	0.024 (2)	−0.0086 (17)	0.0066 (18)	−0.0047 (16)
C6	0.031 (2)	0.027 (2)	0.027 (2)	−0.0016 (17)	0.0060 (18)	−0.0024 (17)
C7	0.027 (2)	0.027 (2)	0.027 (2)	0.0016 (18)	0.0044 (18)	0.0041 (18)
C8	0.038 (3)	0.035 (2)	0.031 (2)	−0.005 (2)	0.0046 (19)	−0.0005 (19)
C9	0.039 (3)	0.049 (3)	0.035 (2)	−0.003 (2)	−0.002 (2)	−0.015 (2)
C10	0.018 (2)	0.028 (2)	0.0198 (19)	0.0017 (16)	0.0014 (16)	0.0021 (16)
C11	0.019 (2)	0.029 (3)	0.0276 (19)	−0.0002 (17)	−0.0004 (15)	0.0009 (17)
C12	0.028 (2)	0.027 (2)	0.027 (2)	0.0033 (18)	0.0015 (18)	−0.0017 (19)
C13	0.028 (3)	0.036 (3)	0.024 (2)	0.0086 (18)	0.0015 (18)	0.0081 (17)
C14	0.017 (2)	0.037 (3)	0.026 (2)	0.0036 (18)	0.0031 (17)	−0.0017 (18)
C15	0.031 (2)	0.028 (2)	0.031 (2)	−0.0088 (18)	0.0038 (19)	−0.0014 (18)
C16	0.031 (2)	0.026 (2)	0.024 (2)	−0.0069 (18)	−0.0004 (18)	0.0017 (18)
C17	0.036 (3)	0.050 (3)	0.028 (2)	0.002 (2)	0.0039 (19)	−0.0027 (19)
C18	0.039 (3)	0.046 (3)	0.029 (2)	−0.006 (2)	0.0012 (19)	0.0016 (18)
C19	0.029 (2)	0.022 (2)	0.0214 (18)	0.0049 (16)	0.0014 (17)	0.0037 (15)
C20	0.017 (2)	0.022 (2)	0.0226 (18)	−0.0001 (15)	−0.0032 (16)	0.0025 (15)
C21	0.020 (2)	0.024 (2)	0.0280 (19)	0.0021 (17)	0.0049 (17)	0.0073 (17)
C22	0.0187 (18)	0.0163 (17)	0.0246 (16)	0.004 (2)	0.0018 (14)	0.0028 (19)
C23	0.022 (2)	0.022 (2)	0.0227 (17)	0.0010 (16)	0.0005 (15)	0.0017 (16)
C24	0.016 (2)	0.030 (2)	0.031 (2)	−0.0019 (16)	0.0030 (17)	0.0035 (17)
C25	0.037 (3)	0.029 (2)	0.027 (2)	0.0041 (18)	0.0047 (18)	0.0051 (17)
C26	0.037 (3)	0.034 (2)	0.039 (2)	−0.0002 (18)	0.0039 (19)	0.0044 (17)
C27	0.030 (2)	0.032 (2)	0.028 (2)	0.0020 (17)	0.0091 (18)	0.0004 (16)
C28	0.042 (3)	0.034 (3)	0.035 (2)	0.0009 (19)	0.0086 (19)	−0.0086 (18)
C29	0.025 (2)	0.019 (2)	0.0238 (18)	0.0002 (16)	−0.0024 (17)	0.0000 (15)
C30	0.027 (2)	0.023 (2)	0.026 (2)	0.0008 (16)	0.0018 (18)	0.0021 (16)
C31	0.021 (2)	0.024 (2)	0.030 (2)	−0.0013 (17)	0.0017 (17)	0.0118 (18)
C32	0.0188 (18)	0.0192 (16)	0.0220 (16)	0.004 (2)	0.0009 (14)	0.0044 (19)
C33	0.019 (2)	0.019 (2)	0.0200 (17)	−0.0017 (15)	−0.0019 (15)	−0.0002 (14)
C34	0.017 (2)	0.020 (2)	0.030 (2)	0.0027 (16)	0.0058 (16)	0.0038 (16)
C35	0.031 (2)	0.025 (2)	0.029 (2)	0.0040 (17)	0.0035 (17)	−0.0004 (17)
C36	0.044 (3)	0.028 (2)	0.033 (2)	−0.0021 (19)	0.000 (2)	0.0016 (17)
C37	0.028 (2)	0.028 (2)	0.0269 (19)	−0.0017 (17)	0.0108 (17)	−0.0049 (17)
C38	0.043 (3)	0.038 (2)	0.039 (2)	0.000 (2)	0.000 (2)	−0.0095 (19)

### Geometric parameters (Å, °)

**Table:**

Co1—O1	2.057 (2)	C14—C17	1.494 (5)
Co1—O3	2.055 (2)	C15—C16	1.356 (5)
Co1—O7	2.117 (3)	C15—H15	0.9500
Co1—O8	2.114 (3)	C16—H16	0.9500
Co1—N1	2.117 (3)	C17—C18	1.501 (5)
Co1—N3	2.120 (3)	C17—H17A	0.9900
O1—C1	1.252 (4)	C17—H17B	0.9900
O2—C1	1.245 (4)	C18—H18A	0.9800
O3—C10	1.262 (4)	C18—H18B	0.9800
O4—C10	1.243 (4)	C18—H18C	0.9800
O5—C24	1.232 (4)	C19—C20	1.376 (5)
O6—C34	1.235 (4)	C19—H19	0.9500
O7—H71	0.840 (19)	C20—C21	1.372 (5)
O7—H72	0.850 (19)	C20—H20	0.9500
O8—H81	0.833 (19)	C21—C22	1.376 (5)
O8—H82	0.857 (18)	C21—H21	0.9500
N1—C23	1.323 (4)	C22—C23	1.370 (4)
N1—C19	1.332 (4)	C22—C24	1.485 (5)
N2—C24	1.342 (4)	C23—H23	0.9500
N2—C25	1.447 (5)	C25—C26	1.511 (5)
N2—C27	1.454 (4)	C25—H25A	0.9900
N3—C29	1.336 (4)	C25—H25B	0.9900
N3—C33	1.326 (4)	C26—H26A	0.9800
N4—C34	1.331 (4)	C26—H26B	0.9800
N4—C35	1.462 (4)	C26—H26C	0.9800
N4—C37	1.452 (4)	C27—C28	1.507 (5)
C1—C2	1.493 (5)	C27—H27A	0.9900
C2—C3	1.379 (5)	C27—H27B	0.9900
C2—C7	1.374 (6)	C28—H28A	0.9800
C3—C4	1.378 (5)	C28—H28B	0.9800
C3—H3	0.9500	C28—H28C	0.9800
C4—C5	1.385 (5)	C29—C30	1.362 (5)
C4—H4	0.9500	C29—H29	0.9500
C5—C6	1.364 (5)	C30—C31	1.380 (5)
C5—C8	1.507 (5)	C30—H30	0.9500
C6—C7	1.362 (5)	C31—C32	1.382 (5)
C6—H6	0.9500	C31—H31	0.9500
C7—H7	0.9500	C32—C33	1.375 (4)
C8—C9	1.496 (5)	C32—C34	1.485 (5)
C8—H8A	0.9900	C33—H33	0.9500
C8—H8B	0.9900	C35—C36	1.509 (5)
C9—H9A	0.9800	C35—H35A	0.9900
C9—H9B	0.9800	C35—H35B	0.9900
C9—H9C	0.9800	C36—H36A	0.9800
C10—C11	1.494 (5)	C36—H36B	0.9800
C11—C12	1.382 (5)	C36—H36C	0.9800
C11—C16	1.383 (5)	C37—C38	1.511 (5)
C12—C13	1.371 (5)	C37—H37A	0.9900
C12—H12	0.9500	C37—H37B	0.9900
C13—C14	1.384 (5)	C38—H38A	0.9800
C13—H13	0.9500	C38—H38B	0.9800
C14—C15	1.376 (5)	C38—H38C	0.9800
			
O3—Co1—O1	177.78 (12)	C18—C17—H17B	109.0
O3—Co1—O8	92.05 (11)	H17A—C17—H17B	107.8
O1—Co1—O8	89.93 (10)	C17—C18—H18A	109.5
O3—Co1—O7	88.68 (11)	C17—C18—H18B	109.5
O1—Co1—O7	89.33 (10)	H18A—C18—H18B	109.5
O8—Co1—O7	179.20 (9)	C17—C18—H18C	109.5
O3—Co1—N1	91.03 (9)	H18A—C18—H18C	109.5
O1—Co1—N1	88.11 (9)	H18B—C18—H18C	109.5
O8—Co1—N1	85.68 (10)	N1—C19—C20	122.9 (3)
O7—Co1—N1	94.00 (10)	N1—C19—H19	118.5
O3—Co1—N3	86.36 (9)	C20—C19—H19	118.5
O1—Co1—N3	94.47 (9)	C21—C20—C19	118.4 (3)
O8—Co1—N3	94.95 (10)	C21—C20—H20	120.8
O7—Co1—N3	85.40 (10)	C19—C20—H20	120.8
N1—Co1—N3	177.33 (11)	C20—C21—C22	119.3 (3)
C1—O1—Co1	127.5 (2)	C20—C21—H21	120.3
C10—O3—Co1	127.3 (2)	C22—C21—H21	120.3
Co1—O7—H71	120 (3)	C23—C22—C21	118.0 (3)
Co1—O7—H72	97 (3)	C23—C22—C24	118.2 (3)
H71—O7—H72	103 (4)	C21—C22—C24	123.7 (3)
Co1—O8—H81	134 (3)	N1—C23—C22	123.7 (3)
Co1—O8—H82	98 (3)	N1—C23—H23	118.1
H81—O8—H82	97 (4)	C22—C23—H23	118.1
C23—N1—C19	117.5 (3)	O5—C24—N2	122.9 (3)
C23—N1—Co1	121.5 (2)	O5—C24—C22	119.7 (3)
C19—N1—Co1	120.9 (2)	N2—C24—C22	117.4 (3)
C24—N2—C25	123.9 (3)	N2—C25—C26	112.1 (3)
C24—N2—C27	117.9 (3)	N2—C25—H25A	109.2
C25—N2—C27	117.0 (3)	C26—C25—H25A	109.2
C33—N3—C29	117.0 (3)	N2—C25—H25B	109.2
C33—N3—Co1	121.0 (2)	C26—C25—H25B	109.2
C29—N3—Co1	121.7 (2)	H25A—C25—H25B	107.9
C34—N4—C37	118.6 (3)	C25—C26—H26A	109.5
C34—N4—C35	123.4 (3)	C25—C26—H26B	109.5
C37—N4—C35	117.7 (3)	H26A—C26—H26B	109.5
O2—C1—O1	125.6 (3)	C25—C26—H26C	109.5
O2—C1—C2	117.5 (3)	H26A—C26—H26C	109.5
O1—C1—C2	116.8 (3)	H26B—C26—H26C	109.5
C7—C2—C3	118.3 (3)	N2—C27—C28	112.2 (3)
C7—C2—C1	121.4 (3)	N2—C27—H27A	109.2
C3—C2—C1	120.3 (3)	C28—C27—H27A	109.2
C4—C3—C2	120.5 (4)	N2—C27—H27B	109.2
C4—C3—H3	119.7	C28—C27—H27B	109.2
C2—C3—H3	119.7	H27A—C27—H27B	107.9
C3—C4—C5	120.7 (4)	C27—C28—H28A	109.5
C3—C4—H4	119.6	C27—C28—H28B	109.5
C5—C4—H4	119.6	H28A—C28—H28B	109.5
C6—C5—C4	117.7 (4)	C27—C28—H28C	109.5
C6—C5—C8	121.0 (3)	H28A—C28—H28C	109.5
C4—C5—C8	121.2 (4)	H28B—C28—H28C	109.5
C7—C6—C5	121.9 (4)	N3—C29—C30	123.6 (3)
C7—C6—H6	119.0	N3—C29—H29	118.2
C5—C6—H6	119.0	C30—C29—H29	118.2
C6—C7—C2	120.8 (4)	C29—C30—C31	118.8 (3)
C6—C7—H7	119.6	C29—C30—H30	120.6
C2—C7—H7	119.6	C31—C30—H30	120.6
C9—C8—C5	112.5 (3)	C30—C31—C32	118.6 (3)
C9—C8—H8A	109.1	C30—C31—H31	120.7
C5—C8—H8A	109.1	C32—C31—H31	120.7
C9—C8—H8B	109.1	C33—C32—C31	118.2 (3)
C5—C8—H8B	109.1	C33—C32—C34	118.4 (3)
H8A—C8—H8B	107.8	C31—C32—C34	123.1 (3)
C8—C9—H9A	109.5	N3—C33—C32	123.8 (3)
C8—C9—H9B	109.5	N3—C33—H33	118.1
H9A—C9—H9B	109.5	C32—C33—H33	118.1
C8—C9—H9C	109.5	O6—C34—N4	122.0 (3)
H9A—C9—H9C	109.5	O6—C34—C32	119.0 (3)
H9B—C9—H9C	109.5	N4—C34—C32	119.1 (3)
O4—C10—O3	124.4 (3)	N4—C35—C36	111.6 (3)
O4—C10—C11	119.9 (3)	N4—C35—H35A	109.3
O3—C10—C11	115.7 (3)	C36—C35—H35A	109.3
C12—C11—C16	119.0 (3)	N4—C35—H35B	109.3
C12—C11—C10	120.7 (3)	C36—C35—H35B	109.3
C16—C11—C10	120.3 (3)	H35A—C35—H35B	108.0
C13—C12—C11	119.5 (4)	C35—C36—H36A	109.5
C13—C12—H12	120.2	C35—C36—H36B	109.5
C11—C12—H12	120.2	H36A—C36—H36B	109.5
C12—C13—C14	121.6 (4)	C35—C36—H36C	109.5
C12—C13—H13	119.2	H36A—C36—H36C	109.5
C14—C13—H13	119.2	H36B—C36—H36C	109.5
C15—C14—C13	117.9 (4)	N4—C37—C38	112.7 (3)
C15—C14—C17	120.8 (4)	N4—C37—H37A	109.1
C13—C14—C17	121.3 (4)	C38—C37—H37A	109.1
C16—C15—C14	121.3 (4)	N4—C37—H37B	109.1
C16—C15—H15	119.4	C38—C37—H37B	109.1
C14—C15—H15	119.4	H37A—C37—H37B	107.8
C15—C16—C11	120.7 (4)	C37—C38—H38A	109.5
C15—C16—H16	119.6	C37—C38—H38B	109.5
C11—C16—H16	119.6	H38A—C38—H38B	109.5
C14—C17—C18	112.9 (3)	C37—C38—H38C	109.5
C14—C17—H17A	109.0	H38A—C38—H38C	109.5
C18—C17—H17A	109.0	H38B—C38—H38C	109.5
C14—C17—H17B	109.0		
			
O8—Co1—O1—C1	28.4 (3)	C12—C13—C14—C17	−176.6 (3)
O7—Co1—O1—C1	−151.3 (3)	C13—C14—C15—C16	−0.6 (6)
N1—Co1—O1—C1	−57.3 (3)	C17—C14—C15—C16	176.8 (4)
N3—Co1—O1—C1	123.3 (3)	C14—C15—C16—C11	−0.7 (6)
O8—Co1—O3—C10	144.3 (3)	C12—C11—C16—C15	1.7 (6)
O7—Co1—O3—C10	−36.0 (3)	C10—C11—C16—C15	179.4 (3)
N1—Co1—O3—C10	−129.9 (3)	C15—C14—C17—C18	−87.5 (5)
N3—Co1—O3—C10	49.5 (3)	C13—C14—C17—C18	89.8 (5)
O3—Co1—N1—C23	143.4 (3)	C23—N1—C19—C20	1.1 (5)
O1—Co1—N1—C23	−34.6 (2)	Co1—N1—C19—C20	−175.4 (2)
O8—Co1—N1—C23	−124.6 (2)	N1—C19—C20—C21	0.4 (5)
O7—Co1—N1—C23	54.6 (2)	C19—C20—C21—C22	−0.6 (5)
O3—Co1—N1—C19	−40.3 (2)	C20—C21—C22—C23	−0.6 (5)
O1—Co1—N1—C19	141.7 (2)	C20—C21—C22—C24	−176.3 (3)
O8—Co1—N1—C19	51.7 (2)	C19—N1—C23—C22	−2.4 (5)
O7—Co1—N1—C19	−129.1 (2)	Co1—N1—C23—C22	174.0 (3)
O3—Co1—N3—C33	41.8 (2)	C21—C22—C23—N1	2.2 (5)
O1—Co1—N3—C33	−140.3 (2)	C24—C22—C23—N1	178.1 (3)
O8—Co1—N3—C33	−49.9 (2)	C25—N2—C24—O5	167.0 (3)
O7—Co1—N3—C33	130.8 (2)	C27—N2—C24—O5	0.2 (5)
O3—Co1—N3—C29	−131.8 (2)	C25—N2—C24—C22	−13.5 (5)
O1—Co1—N3—C29	46.1 (3)	C27—N2—C24—C22	179.7 (3)
O8—Co1—N3—C29	136.4 (2)	C23—C22—C24—O5	−65.9 (4)
O7—Co1—N3—C29	−42.9 (2)	C21—C22—C24—O5	109.7 (4)
Co1—O1—C1—O2	−26.8 (5)	C23—C22—C24—N2	114.6 (4)
Co1—O1—C1—C2	152.4 (2)	C21—C22—C24—N2	−69.8 (4)
O2—C1—C2—C7	174.6 (3)	C24—N2—C25—C26	111.8 (4)
O1—C1—C2—C7	−4.6 (5)	C27—N2—C25—C26	−81.3 (4)
O2—C1—C2—C3	−2.9 (5)	C24—N2—C27—C28	87.8 (4)
O1—C1—C2—C3	177.8 (3)	C25—N2—C27—C28	−79.9 (4)
C7—C2—C3—C4	1.9 (6)	C33—N3—C29—C30	−1.3 (5)
C1—C2—C3—C4	179.5 (4)	Co1—N3—C29—C30	172.6 (3)
C2—C3—C4—C5	−2.4 (7)	N3—C29—C30—C31	0.2 (5)
C3—C4—C5—C6	1.4 (7)	C29—C30—C31—C32	0.4 (5)
C3—C4—C5—C8	178.6 (3)	C30—C31—C32—C33	0.1 (5)
C4—C5—C6—C7	−0.1 (7)	C30—C31—C32—C34	173.7 (3)
C8—C5—C6—C7	−177.2 (4)	C29—N3—C33—C32	1.8 (5)
C5—C6—C7—C2	−0.3 (6)	Co1—N3—C33—C32	−172.1 (2)
C3—C2—C7—C6	−0.6 (6)	C31—C32—C33—N3	−1.3 (5)
C1—C2—C7—C6	−178.2 (3)	C34—C32—C33—N3	−175.2 (3)
C6—C5—C8—C9	67.8 (5)	C37—N4—C34—O6	2.1 (5)
C4—C5—C8—C9	−109.3 (5)	C35—N4—C34—O6	−171.1 (3)
Co1—O3—C10—O4	29.5 (5)	C37—N4—C34—C32	−178.4 (3)
Co1—O3—C10—C11	−150.4 (2)	C35—N4—C34—C32	8.4 (5)
O4—C10—C11—C12	3.4 (5)	C33—C32—C34—O6	66.2 (4)
O3—C10—C11—C12	−176.7 (3)	C31—C32—C34—O6	−107.4 (4)
O4—C10—C11—C16	−174.2 (3)	C33—C32—C34—N4	−113.3 (4)
O3—C10—C11—C16	5.6 (5)	C31—C32—C34—N4	73.1 (4)
C16—C11—C12—C13	−1.5 (6)	C34—N4—C35—C36	−109.6 (4)
C10—C11—C12—C13	−179.2 (3)	C37—N4—C35—C36	77.2 (4)
C11—C12—C13—C14	0.3 (6)	C34—N4—C37—C38	−92.0 (4)
C12—C13—C14—C15	0.8 (7)	C35—N4—C37—C38	81.6 (4)

### Hydrogen-bond geometry (Å, °)

**Table:**

*D*—H···*A*	*D*—H	H···*A*	*D*···*A*	*D*—H···*A*
O7—H71···O6^i^	0.84 (3)	2.02 (4)	2.767 (4)	148 (3)
O7—H72···O4	0.85 (2)	1.79 (2)	2.622 (3)	167 (4)
O8—H81···O4^ii^	0.83 (4)	1.98 (3)	2.811 (3)	176 (4)
O8—H82···O2	0.86 (2)	1.77 (2)	2.614 (3)	167 (5)
C15—H15···O5^iii^	0.95	2.40	3.170 (5)	138
C20—H20···O4^iii^	0.95	2.49	3.407 (4)	163
C30—H30···O6^iv^	0.95	2.34	3.262 (4)	163

Symmetry codes: (i) −*x*, *y*−1/2, −*z*+2; (ii) −*x*, *y*+1/2, −*z*+2; (iii) −*x*+1, *y*+1/2, −*z*+2; (iv) −*x*−1, *y*−1/2, −*z*+2.
